# Interrater reliability of routine screening for risk of malnutrition with the Mini Nutritional Assessment Short-Form in hospital

**DOI:** 10.1038/s41430-022-01080-y

**Published:** 2022-02-22

**Authors:** Ulrike Sonja Trampisch, Maryam Pourhassan, Diana Daubert, Dorothee Volkert, Rainer Wirth

**Affiliations:** 1 Department of Geriatric Medicine, Marien Hospital Herne, Ruhr University Bochum, Herne, Germany; 2 Institute for Biomedicine of Aging, Friedrich-Alexander-Universität Erlangen-Nürnberg, Nürnberg, Germany

**Keywords:** Malnutrition, Epidemiology

## Abstract

**Background/objectives:**

The Mini Nutritional Assessment Short-Form (MNA-SF) is the recommended screening tool for older persons. Data on interrater reliability in clinical routine are rare. Thus, we wanted to quantify the interrater reliability of the MNA-SF in hospital.

**Subjects/methods:**

This observational cross-sectional study was undertaken retrospectively. The study population comprised 105 participants. Risk of malnutrition was measured twice with the routine MNA-SF performed by nurses (within 24 h after admission) and a dedicated dietician (one to three days after the first MNA-SF). The MNA-SF score was analyzed for interrater reliability between nurse and dietician.

**Results:**

Participants’ mean age was 82.4 (±7.1) years and 71 (68%) were women. The mean total MNA-SF score was 7.4 (±2.4) assessed by dietician and 7.8 (±2.3) assessed by nurse. The intra-class correlation coefficient between the total MNA-SF scores was 0.74 (0.61; 0.82), indicating moderate reliability. For the MNA-SF nutritional status, Cohens Kappa was 0.37 (*p* < 0.05) showing a fair agreement.

**Conclusion:**

Multiple misclassifications were observed between malnutrition and risk of malnutrition. Because mean scores were near the border between malnutrition and risk of malnutrition, we recommend to consider the total MNA-SF score in addition to the three risk groups to assess nutritional risk in geriatric hospital patients.

## Introduction

The prevalence of malnutrion in geriatric hospital patients is high. Malnutrition has serious consequences for health outcomes [1]. Malnourished patients show e.g., higher complication rates, such as health care associated infections, increased length of hospital stay, unfavorable rehabilitation outcome and higher risk of mortality. Many trials proved, that treating malnutrition is capable to improve health outcomes [2–4]. The high prevalence of malnutrition and the treatment effectiveness justify the recommendation to integrate screening for risk of malnutrition into the geriatric assessment [5]. In this regard, the Mini-Nutritional-Assessment (MNA) is the recommended tool for older patients, for whom it has been specifically validated [6]. One of the problems of the full MNA is the fact that it takes about 15−20 min, which is not realistic for routine screening in hospitals, nursing homes and in the home care area. Therefore, the first six screening questions of the 18 items of the full form have been validated as a short form (SF). The results of the MNA-SF do not substantially differ from the results of the full form [7]. The MNA-SF takes only very few minutes to be performed and is therefore acceptable and practical for clinical routine. However, a reduction of items may lead to more susceptibility for errors, because the impact of every single question increases. Studies under research conditions found excellent test-retest reliability [8–10]. However, data on interrater reliability are rare, especially from clinical routine. Coincidentally, we observed during an ongoing clinical trial (Optimizing refeeding in malnourished frail older patients, DRKS-ID: DRKS00017324; https://www.drks.de/drks_web/setLocale_EN.do) that the German MNA-SF nutritional status performed by a dietician (nutritional specialist) frequently differed from the one that has been performed in clinical routine by the nurses on the ward. We noticed the lack of evidence regarding the interrater reliability of screening for risk of malnutrition using MNA-SF in routine geriatric hospital setting. Thus, we wanted to quantify the interrater reliability between the screening results for risk of malnutrition with MNA-SF performed by the nurse and the dietician, differentiated by the total MNA-SF score and the categories of MNA-SF nutritional status. Moreover, we investigated if single items of the MNA-SF are especially vulnerable for errors.

## Subjects and methods

This observational cross-sectional study was undertaken retrospectively between July 2020 and October 2020 in the acute care geriatric hospital department of the university hospital Marien Hospital Herne, Germany. The MNA-SF is part of the routine geriatric assessment in the geriatric department since many years. The study population comprised 105 older participants. Participants were consecutively admitted to the geriatric acute care ward. Inclusion criteria were admission to the acute care geriatric hospital department, 60 years or older, and two screening results for malnutrition with MNA-SF performed by nurse and dietician. Exclusion criteria were severe cognitive impairment and palliative situation. Data were extracted from medical records. The study protocol had been approved by the ethical committee of Ruhr University Bochum (no 20-7077-BR approved on 29.10.2020). The study is registered at German Clinical trial register (DRKS-ID: DRKS00024658; https://www.drks.de/drks_web/setLocale_EN.do).

Geriatric assessment was routinely performed at hospital admission including (among other things) patients’ date of birth, sex, height, and weight. Body weight was assessed in light clothing on a seat scale with an accuracy of 0.1 kg, and height was measured to the nearest 0.5 cm with a stadiometer during hospitalization. If the measurement of weight and/or height was not possible, weight and/or height were estimated based on the patient’s report. Activities of daily living were determined using the Barthel-Index (BI) [11]. The point’s range for the German version of the BI is 0–100 pts, with 100 pts indicating independence in all activities of daily living. Cognitive function was measured with the Montreal Cognitive Assessment (MoCA) [12] in its translated German version. The point’s range for the German version of the MoCA is 0–30 pts, with higher pts. indicating normal cognitive function.

Risk of malnutrition was measured according to the MNA-SF [7], which is a validated tool for the screening for risk of malnutrition of geriatric patients across settings. We used the translated German version of the MNA-SF, which was not especially validated after translation. In our hospital, nurses receive individual unstructured training how to perform the MNA-SF from a more experienced nurse. MNA-SF nutritional status was assessed twice, each in a personal interview with the patient. A nurse performed the routine screening with MNA-SF first within 24 h after hospital admission. The screening by nurse was carried out by different nurses, depending on who was on duty at the ward at the day of admission. A dietician (always the same person) performed the second screening with MNA-SF one to three days after the first screening. In the presented paper following, screening by dietician is assumed the “expert” opinion.

The MNA-SF consists of six items (A–F) with a scoring up to 3 points per item: (A) Has food intake declined over the past 3 months due to loss of appetite, digestive problems, chewing or swallowing difficulties? (B) Involuntary weight loss during the last 3 months? (C) Mobility? (D) Has the patient suffered psychological stress or acute disease in the past 3 months? (E) Neuropsychological problems? (F1) Body Mass Index (BMI) (F2) Calf circumference (CC) in cm. BMI (F1) was read off a BMI disc (nurse) or calculated (dietician). If height or weight could not be captured, calf circumference (CC) in cm was measured and categorized as CC < 31 or ≥31 instead, as recommend by the MNA-SF. The screening result of the MNA-SF is the calculated total MNA-SF score (0–14). The total MNA-SF score is then transferred to the corresponding MNA-SF nutritional status (categories: normal nutritional status [12–14], risk of malnutrition [8–11], malnourished (0–7)).

The statistical analysis was completed using SPSS statistical software (SPSS Statistics for Windows, 137 IBM Corp, Version 27.0, Armonk, NY, USA). Means and standard deviations (SDs) were used for continuous data. Categorical variables are demonstrated as *n* (%). Participants were divided into three groups according to the MNA-SF classification (malnourished, risk of malnutrition and normal nutritional status). Participant’s age was calculated for the day of hospital admission. If the assessment with MoCA could not be fully recorded for non-cognitive reasons, the result was extrapolated according to the questions answered.

In the present work, the MNA-SF score was analyzed for interrater reliability between routine screening for risk of malnutrition with MNA-SF of the nurse and of the dietician in two analyses: interrater reliability for the total MNA-SF score (interval-scaled) and for the MNA-SF nutritional status (categorical variable).

A Bland-Altman plot was used to graphically demonstrate the consistency of the total MNA-SF score. The Bland-Altman plot compares graphically the two total MNA-SF scores (values 0–14). It also shows possible relationships between measurement inaccuracies (estimated via the differences between the two total MNA-SF scores) with the level of the true value (estimated via the mean value of the two total MNA-SF scores) [13]. Therefore, the Bland-Altman plot allows identification of any systematic difference between the measurements or possible outliers. 95% limits of agreement for each comparison (average difference ± 1.96 standard deviation of the difference) were computed. To calculate the interrater reliability between the interval-scaled total MNA-SF score of the nurse and the dietician, the intra-class correlation coefficient (ICC) estimates and their 95% confident intervals were used in the variant ICC (model two-way mixed, absolute agreement) [14]. An ICC of 1 indicates excellent interrater reliability, whereas an ICC of 0 indicates no interrater reliability [15].

Cohens Kappa was used to assess the interrater reliability between the categorical MNA-SF nutritional status of the nurse and the dietician. It was calculated for the MNA-SF nutritional status (normal nutritional status, risk of malnutrition, malnourished) and for each single item (A–F). A Kappa of 1 indicates perfect agreement, whereas a kappa of 0 indicates agreement equivalent to chance. A *p* < 0.05 was considered as the limit of significance.

## Results

The study population comprised 105 older participants aged between 62 and 97 years (mean age 82.4 ± 7.1 years) with 68% being women. Reasons for hospital admission in this multimorbid and frail cohort were predominantly falls, fractures, infections, cardiovascular and neurodegenerative disease. Participants had a mean MoCA of 17.7 (±5.9) and a mean BI of 47.4 (±16.7). All baseline characteristics of study participants are summarized in Table 1.

**Table 1:** Characteristics of the study participants in total (*n* = 105).

	Missing values	*n* (%) or mean (SD)
Age (years)	0	82.4 ± 7.1
Sex	0	
Female		71 (67.6%)
Male		34 (32.4%)
Height (cm)	1	166 ± 9.1
Weight (kg)	1	70.1 ± 16.6
BMI	0	25.3 ± 5.4
Calf circumference in cm	103	
<31 cm		1 (1.0%)
≥31 cm		1 (1.0%)
MoCA	25	17.7 ± 5.9
Barthel-Index	0	47.4 ± 16.7
MNA-SF score nurse	0	7.8 ± 2.3
MNA-SF score dietician	0	7.4 ± 2.4
difference score nurse-dietician	0	0.3 ± 2.1
MNA-SF nutritional status nurse	0	
Normal nutritional status		3 (2.9%)
Risk of malnutrition		62 (59.0%)
Malnourished		40 (38.1 %)
MNA-SF nutritional status dietician		
Normal nutritional status		3 (2.9%)
Risk of malnutrition		51 (48.6%)
Malnourished		51 (48.6%)

*SD* standard deviation, *cm* centimeter, *kg* kilogram, *BMI* body mass index, *MoCA* Montreal Cognitive Assessment, *MNA-SF* Mini Nutritional Assessment Short-Form.

### MNA-SF total score and interrater reliability

**Fig. 1:**
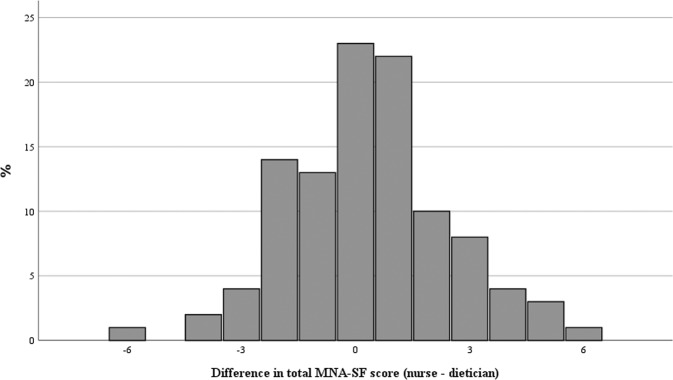
Frequency of difference in total MNA-SF score (nurse - dietician). MNA-SF Mini Nutritional Assessment Short-Form.

The mean total MNA-SF score was 7.4 (±2.4) assessed by dietician with a range from 2 to 12. The mean total MNA-SF score was 7.8 (±2.3) assessed by nurse with a range from 2 to 13. The mean difference of the total MNA-SF score (nurse - dietician) was 0.3 (±2.1) ranging from −6 to 6. The highest frequency of differences was 0 in 21.9% of patients, followed by 1 with 21.0% of patients (Fig. 1).

**Fig. 2:**
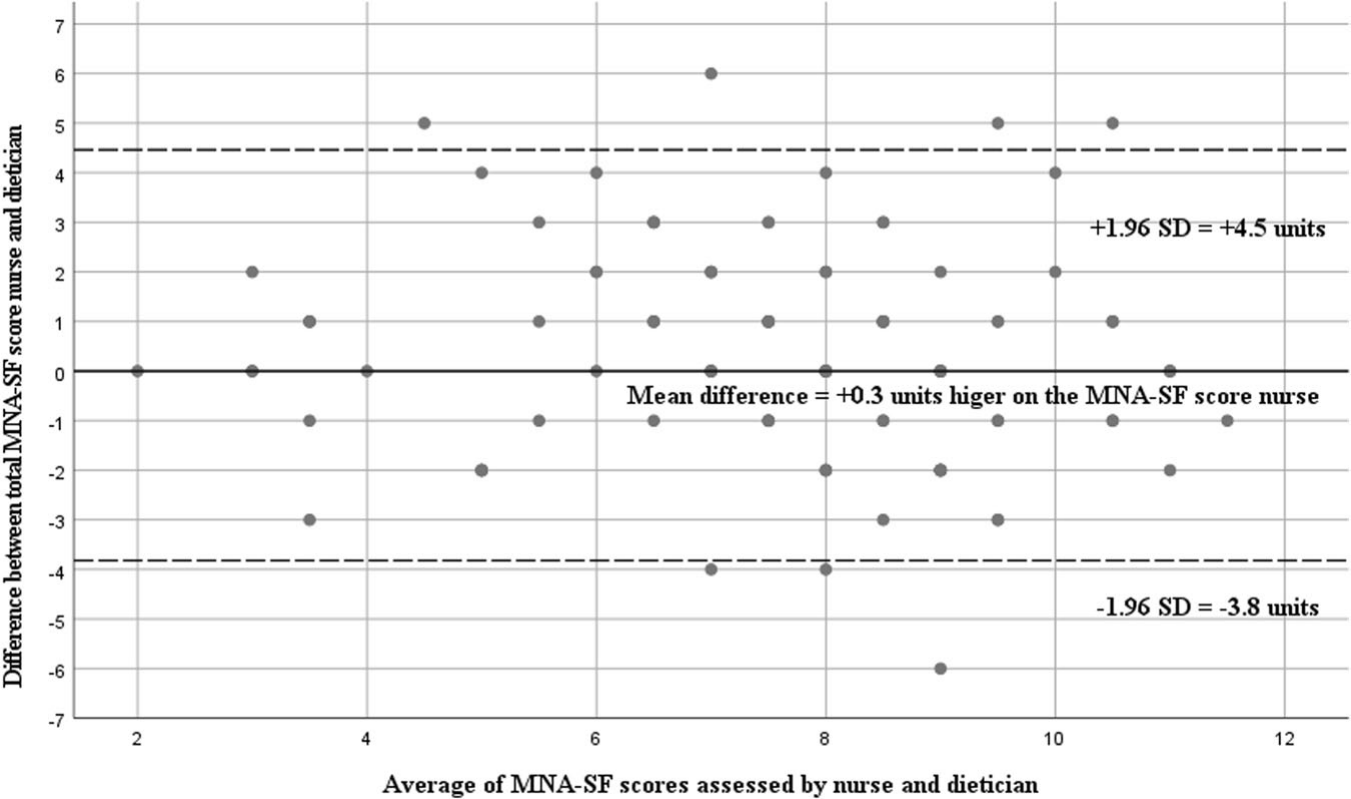
Bland-Altman plot agreement between total MNA-SF score of nurse and dietician. MNA-SF Mini Nutritional Assessment Short-Form.

The 95% limits of agreement for the Bland-Altman plot were 4.4 (=0.3 + 2.1*1.96) and −3.8 (=0.3 − 2.1*1.96). The Bland-Altman plot shows no systematic bias (e.g., higher range with increasing total MNA-SF score) between total difference and mean value of the two total MNA-SF scores (Fig. 2). The mean difference (+0.34) between the two total MNA-SF scores indicates that there are minor deviations, visible from the solid line near 0 in the Bland-Altman plot. Measurements are equally distributed in between upper and lower limits of agreement, visible from the dashed lines. The estimate for the ICC with 95% confidence interval between the total MNA-SF score of the nurse and the total MNA-SF score dietician was 0.74 (0.61; 0.82), indicating moderate reliability [15].

### MNA-SF nutritional status and interrater reliability

**Fig. 3:**
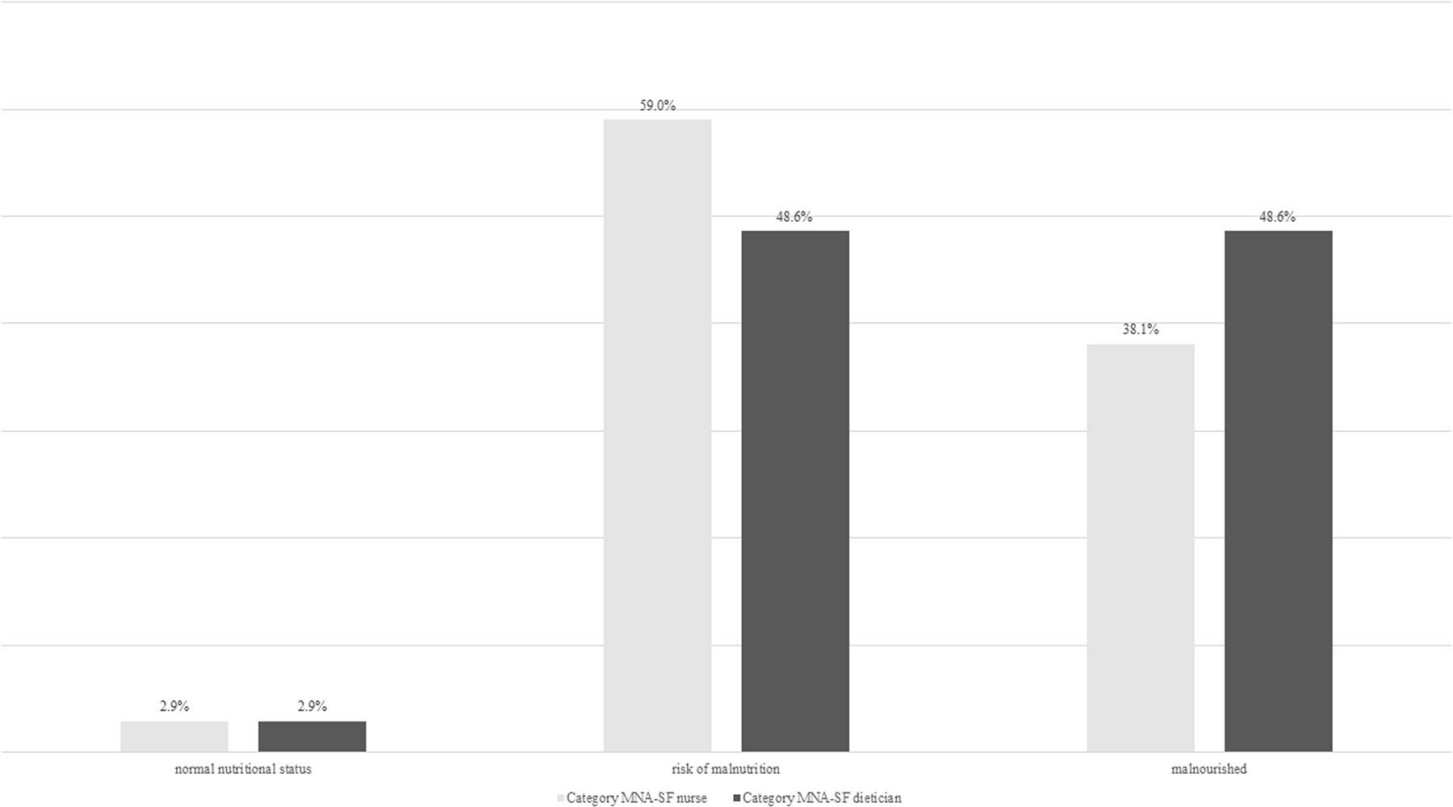
Frequency of MNA-SF category of nurse and dietician. MNA-SF Mini Nutritional Assessment Short-Form.

According to MNA-SF nutritional status assessed by the dietician, 3 (2.9%) had a normal nutritional status, 51 (48.6%) were at risk of malnutrition, and 51 (48.6%) were malnourished. Nurses categorized 3 (2.9%) having a normal nutritional status (±0% compared to dietician’s nutritional status), 62 (59.0%) were at risk of malnutrition (+9.2% compared to dietician’s nutritional status), 40 (38.1%) malnourished (−10.5% compared to dietician’s nutritional status) (Fig. 3). The BMI of two patients could not be calculated by nurse due to missing weight and height (MNA-SF item F1), therefore CC was measured (MNA-SF item F2). The dietician calculated BMI of all patients. For the MNA-SF nutritional status, Cohens Kappa was 0.37 (*p* < 0.05) showing a fair agreement between MNA-SF nutritional status assessed by dietician and nurse [16]. One participant (1%) was categorized by dietician to have a “normal nutritional status”, and the nurse categorized this patient to be “malnourished”. Another participant (1%) was categorized by dietician to be “malnourished”, and the nurse categorized this patient to have a “normal nutritional status” (Table 2). Cohens Kappa for the single items of the MNA-SF (A-F) ranges from 0.21 to 0.69 (Table 3).

**Table 2:** MNA-SF nutritional status MNA-SF dietician and nurse and Cohens Kappa.

		MNA-SF nutritional status dietician				Cohens Kappa
		Normal nutritional status	Risk of malnutrition	Malnourished	Total	
MNA-SF nutritional status nurse	Normal nutritional status	**0 (0%)**	2 (66.7%)	1 (33.3%)	3 (2.9%)	0.37 (*p* < 0.05)
	Risk of malnutrition	2 (3.2%)	**40 (64.5%)**	20 (32.3%)	62 (59.0%)	
	Malnourished	1 (2.5%)	9 (22.5%)	**30 (75.0%)**	40 (38.1%)	
	Total	3 (2.9%)	51 (48.6%)	51 (48.6%)	105	

Agreements are shown in bold.

*MNA-SF* Mini Nutritional Assessment Short-Form.

**Table 3:** Cohens Kappa for single MNA-SF items.

	Question	Cohens Kappa
A	Has food intake declined over the past 3 months due to loss of appetite, digestive problems, chewing or swallowing difficulties?	0.23 (*p* < 0.05)
B	Involuntary weight loss during the last 3 months?	0.21 (*p* < 0.001)
C	Mobility?	0.60 (*p* < 0.001)
D	Has the patient suffered psychological stress or acute disease in the past 3 months?	Not applicable
E	Neuropsychological problems?	0.60 (*p* < 0.001)
F1	Body Mass Index (BMI) (weight in kg)/(height in m^2^)?	0.69 (*p* < 0.001)

*MNA-SF* Mini Nutritional Assessment Short-Form.

## Discussion

We found a fair to moderate interrater reliability for the screening for risk of malnutrition in routine screening in hospital. Overall, this is a satisfactory result and underlines that the screening for the risk of malnutrition can sufficiently be performed by nurses, since they know many characteristics of the patient (e.g., cognition, mobility). In contrast to other studies [8–10], our study represents the actual “real life” routine in hospital, and not an artificially created situation under study conditions. Studies under research conditions examined test-retest reliability. Kather et al. (2020) found excellent test-retest reliability (ICC = 0.93) of the MNA-SF in patients aged 75 years and older after catheter-based interventions undergoing cardiac rehabilitation [8]. These results are comparable to previous research by Bleda et al. (2002) and Lin et al. (2019), who examined the test-retest reliability of the original full MNA in institutionalized older people and patients with stroke, respectively. Bleda et al. (2002) reported an excellent test-retest reliability (ICC = 0.89) and a substantial agreement (Kappa = 0.78) in 67 institutionalized patients [9]. Lin et al. (2019) found excellent test-retest reliability (ICC = 0.91) in test-retest reliability in 59 patients with stroke in the full MNA [10].

In our study, the mean total MNA-SF scores assessed by dietician (7.4) and nurse (7.8) revealed a minor difference of 0.3 showing a moderate interrater reliability. The mean scores, however, are exactly on the border between the two categories “risk of malnutrition” [8–11] and “malnourished” (0–7). This partly explains why the interrater reliability of the MNA-SF nutritional status only shows fair agreement. Although the overall interrater reliability between the assessment by a dedicated dietitian and routine assessment by the nurse in charge was only fair (Cohens Kappa: 0.37 (*p* < 0.05)), the present study demonstrates, that severe misclassification with possible clinical consequences happened rarely. Only one participant, who was classified as malnourished by the dietician was rated as normal nutritional status in routine assessment. The opposite misclassification, which was also seen in one other patient, would barely be harmful for the patient, because it led to nutritional counseling first, where this kind of misclassification would be discovered. Even if oral nutritional supplements were given without further counseling, this would not harm any patient, but lead to avoidable costs. Multiple misclassifications were observed between malnutrition and risk of malnutrition. Their clinical relevance depends on the routine management of patients at risk of malnutrition and is likewise probably low. If there is a clinical suspicion of categorical misclassification, the total MNA-SF score should also be taken into account for nutritional risk grading (particularly if the value is near the borderline between the two categories).

On the level of individual items of the MNA-SF, the first two questions about food intake and weight loss demonstrated the lowest level of agreement, with a Cohens Kappa of 0.23 (*p* < 0.05) and 0.21 (*p* < 0.05). Respectively, pointing out that it possibly would increase accuracy if moderate and severe decrease of food intake would be defined more precisely, as done in other screening tools. To the contrary, the weight loss item is clearly defined but frequently classified as unknown in the routine assessment. In contrast to the nurse in charge, the dedicated dietician often has gathered more information about the nutritional situation of the patient, including current weight and weight loss. The psychological stress/acute disease item should have always been rated as “yes” in this study, since hospitalized patients always suffer an acute disease. About 13% of this item was rated with “no” exclusively by the nurse. Thus, we conclude that nurses need more structured training on correctly performing the screening. In addition, it could be hypothesized that the accuracy of the screening for risk of malnutrition with MNA-SF could be improved by performing it on the second or third day of hospital stay, after obtaining more information about the patient, after body weight has been measured and food intake has been observed for more than one meal. Therefore, we recommend a re-screening after a few days for patients with missing or uncertain data to prevent only relying on the subjective information of the patient.

This study has some limitations. First, it is a single center study with a limited number of patients, which may affect the generalizability of the results. Although our study is a retrospective data analysis and some of the patients were subjects in different studies, the fraction of people being at risk of malnutrition or malnourished are comparable with a consecutively recruited study group [17, 18]. In addition, the routine assessment performed by nurses and the assessment performed by the dedicated dietician were not performed at the same day, so the dietician could have more information about the patient. All of these limitations may cause a bias. On the other hand, this situation reflects best the weakness of routine screening at time of hospital admission. A general problem is that only few patients had a normal nutritional status, which is expected in geriatric hospital patients, but which reduces the chance to detect misclassifications. Furthermore, the order of the screening was not random. The first screening was always carried out by the nurse and the second screening by the dietician. This may lead to a bias, since the patient had time after the first screening to reflect on the questions and to answer them more carefully during the subsequent screening by the dietician. Moreover, the day of the hospital admission is assumed stressful for the patient, and at the time of the screening by the dietician, the patient might be a little bit more used to the situation in the hospital and therefore able to answer the screening questions well considered.

We recommend taking into account the MNA-SF total score in addition to the MNA-SF categories for grading the (the risk of) malnutrition in each category. Moreover, we recommend performing the MNA-SF with sufficient information about the patient, even if screening is postponed to the second or third day of the hospital stay. Overall, nurses need to receive a structured trained in performing the screening for the risk of malnutrition using MNA-SF.

## Data Availability

Additional data are available from the corresponding author on reasonable request.
